# Sputum quality affects assessment of airway microbiology in childhood asthma

**DOI:** 10.1186/s12931-025-03266-x

**Published:** 2025-06-04

**Authors:** Steven L Taylor, Collin R Brooks, Levi Elms, Sarah K Manning, Alyson Richard, Jeroen Burmanje, Jeroen Douwes, Geraint B Rogers

**Affiliations:** 1 Microbiome and Host Health, South Australian Health and Medical Research Institute, North Terrace, Adelaide, SA 5001 Australia; 2 College of Medicine and Public Health, Flinders University, Adelaide, Australia; 3 Research Centre for Hauora and Health, Massey University, Wellington, New Zealand

**Keywords:** Squamous cell, Inflammatory phenotyping, *Haemophilus influenzae*, Sputum plug, Microbiota

## Abstract

**Background:**

The analysis of sputum is the principal basis for characterising lower airway microbiology in those with chronic respiratory conditions. For such analysis to be informative, samples that poorly reflect the lower airways must be identified and removed. Our cross-sectional study explored the relationship between the quality of sputum samples and their microbiological content. We further investigated the impact of excluding low quality samples on observed microbiota-disease relationships in childhood asthma.

**Methods:**

Induced sputum was collected from children with or without asthma. Sputum quality was assessed according to squamous cell%, cell viability%, detection of sputum plugs, and salivary α-amylase levels. Sputum microbiota was characterised by 16S rRNA amplicon sequencing and qPCR.

**Results:**

Of 170 participants, 130 had asthma. Between 19% (32/170) and 29% (53/170) of samples were deemed to be of insufficient quality, depending on the quality criterion applied. Stratification of samples based on any of the sputum quality cut-offs resulted in significant differences in microbiota characteristics (all *p* < 0.05), with salivary α-amylase the least discriminant between microbiota of acceptable and unacceptable samples. The removal of 53 poor-quality samples based on ≥ 30% squamous cells identified a difference in the sputum microbiota by asthma status (*p* = 0.017) that was not evident otherwise, including significantly higher levels of *Haemophilus* and *Gemella* in asthma samples.

**Conclusions:**

Upper airway contamination of induced sputum samples from children is common. Exclusion of samples based on ≥ 30% squamous cells enables identification of asthma-airway microbiology relationships that are otherwise not apparent.

**Supplementary Information:**

The online version contains supplementary material available at 10.1186/s12931-025-03266-x.

## Background

Analysis of sputum underpins our understanding of airway pathophysiology in chronic lung disease. Sputum samples (spontaneous or induced) act as the principal basis for diagnostic microbiology, basic research, disease phenotyping, and clinical management [1, 2]. For example, assessment of sputum leukocyte populations, particularly eosinophil percentage, has been useful in identifying T2 high asthma [3] and thus predicting treatment response to biological agents [4] and inhaled corticosteroids (ICS) [5]. However, despite its utility, sputum is a poorly defined sample type that is compositionally complex and highly variable [6]. Containing mucus, saliva, serous fluid, epithelial and inflammatory cells, microbes, and other components, sputum samples may represent features of both upper (including the buccal cavity) and lower airways [6]. Samples containing material derived from the upper airways may misrepresent investigations of lower airway pathophysiology [7]. As many of the bacteria that are associated with chronic lung disease are common within the oropharynx [8], this presents considerable challenges for the analysis of the lower airway microbiology [9, 10].

Attempts have been made to develop standardised quality criteria for sputum (see below; [1, 11, 12]). However, these criteria are primarily designed to facilitate accurate assessment of leukocyte cell populations. As yet, no standardised approach exists to determine the extent of upper airway contamination, and there is no widespread consensus regarding what constitutes acceptable sputum quality.

Differential (human) cell count characteristics are commonly used as a basis to assess sputum quality, including the proportion of: squamous epithelial cells [10, 13–15] (which are abundant in the upper airways [16]), non-squamous cells such as leukocytes and columnar epithelial cells (which are indicative of the lower airways), and the proportion of viable non-squamous cells [11, 12, 17]. Sputum characteristics, such as the appearance, volume, or number of mucus plugs [6, 18], and quantification of proteins representing specific compartments, such as salivary α-amylase [13, 19, 20] have also been used. While these measures can be informative, cut-off levels used to define sample acceptability either do not exist or differ substantially between studies. For example, the proportion of squamous cells that are considered unacceptable range from 10% [14] to 80% [10, 15]. Additionally, while some airway microbiome studies have used sputum quality assessments to exclude samples containing substantial upper airway contamination [10, 14, 21], no systematic comparison of the performance of different approaches, or their impact on airway microbiota analysis, has been reported.

Our study aimed to investigate the association between quality criteria (i.e. the extent to which samples are representative of the upper and lower airways) and airway microbiota composition in childhood asthma. Childhood asthma was chosen specifically because the modest divergence in airway microbiota characteristics between asthmatic and non-asthmatic individuals makes the potential impact of upper airway contamination more pronounced. Our goal was to identify the assessment criteria that performed best in excluding samples with substantial upper airway contamination, allowing reliable examination of relationships between airway microbiology and disease.

## Methods

### Study population

Detailed clinical assessments and methodology are provided in the online supplement. Participants aged 8–14 years were recruited from Wellington, New Zealand, through community-based recruitment between 2018 and 2020. Asthma was defined as wheezing/whistling in the chest and/or asthma medication use within the last 12 months [22]. Informed consent was obtained from participants/parents in accordance with the Declaration of Helsinki, and the study was approved by the Northern B Health and Disability Ethics Committee (16/NTB/64).

### Spirometry

Spirometry was measured using an Easyone spirometer (NDD Medizintechnik AG, Zurich, Switzerland) as described previously [23].

### Sputum induction and sample processing

Hypertonic saline challenge/sputum induction was conducted as described previously [24]. Sputum plugs (the visibly viscid components of the entire expectorate) were separated and collected using sterile tweezers and displacement pipette. Sputum plug sample characteristics and volume were recorded. An untreated proportion of the sputum sample (including plugs, where available) was aliquoted and stored at -80 °C for subsequent microbiota characterisation and salivary amylase measurement (see below).

### Squamous cell and viability assessment

Sputum plugs were dispersed using dithiothreitol (Sputasol, Oxoid Ltd, Hampshire, England), filtered through a 60 μm filter (Millipore, County Cork, Ireland), centrifuged at 400 × g, and resuspended in phosphate buffered saline (PBS). Total non-squamous cell count (TCC/mL) and viability (using trypan-blue exclusion) were determined using a haemocytometer under light microscopy. The remaining cell suspension was resuspended to 1 × 10^6^ cells/mL and cytospin slides prepared and stained using a Diff-Quik^®^ fixative/stain set (Dade Behring, Deerfield, IL). Squamous and non-squamous cells were counted until a minimum of 400 non-squamous cells were counted. If non-squamous cells could not be counted accurately due to excessive squamous cells, the sample was assigned a squamous percentage of 80%.

### Sputum processing for DNA extraction and salivary α-amylase quantification

Raw, untreated sputum was weighed and 100 mg of was suspended in 300 µL of PBS, vortexed for 10 s, and placed on ice for 2 min. Samples were centrifuged at 13,000 × g for 10 min with the resulting pellet used for DNA extraction and supernatant used for salivary α-amylase quantification. A blank extraction control and a mock community sample containing equal proportions of 10 bacterial species (five Gram-positive, five Gram-negative) were included in each extraction batch.

### Salivary α-amylase measurement

Salivary α-amylase concentration was measured using the Salimetrics salivary α-amylase kinetic enzymatic kit (Salimetrics, PA, USA) as per manufacturer’s instructions. However, rather than a 1:200 sample dilution suggested for saliva, sputum supernatant was diluted at 1:20 to be detectable within assay range. Salivary α-amylase levels were back-calculated per mL of sputum. The adjusted limits of detection were 0.025 to 20 U/mL. Six samples were above the upper limit of detection and were therefore assigned the upper limit of quantification (20 U/mL).

### Sputum quality cut-offs

As no standardised sputum quality criteria exist, we assessed four measures of sputum quality (squamous cell%, cell viability%, sputum plugs, and salivary α-amylase) and applied a cut-off for each measure. For squamous cell percentage, ≥ 30% was considered inadequate, as reported previously [22], with sensitivity analyses utilising cut-offs of 20% and 25%. For cell viability percentage, < 50% was considered inadequate as reported previously [12, 22]. For sputum plugs, absence of visible plugs was considered inadequate. Finally, for salivary α-amylase concentration, ≥ 7.5 U/mL was considered inadequate, representing approximately 5% of the general population average salivary α-amylase concentration [25].

### Sputum microbiota assessment

Detailed descriptions of sputum DNA extraction, bacterial load quantitative PCR (qPCR), 16S rRNA sequencing, and bioinformatic processing are provided in the online supplement. Briefly, DNA was extracted from approximately 100 mg of sputum using a combination of mechanical, heat, chemical, and enzymatic lysis, followed by purification using the EZ-10 Spin Column Genomic DNA Kit (Bio Basic, Inc., Ontario, Canada). Total bacterial load was quantified using primers targeting the 16S rRNA gene, as described previously [26]. 16S rRNA amplicon sequencing was performed targeting the V1-3 region using an Illumina MiSeq. Mock community controls and blank extraction controls were sequenced alongside samples to assess taxon representation and reagent contaminants, respectively. Bioinformatic processing was performed using QIIME2 [27](release 2019.4), using the DADA2 plugin [28], and aligned against the SILVA database (v132). The median clean and filtered read count was 5,210 reads (interquartile range: 4,472, 5,850) and samples with fewer than 2,979 were excluded based on alpha rarefaction curve. Reads have been deposited in the European Bioinformatics Institute European Nucleotide Archive (PRJEB57744).

### Statistical analysis and visualisation

To assess compositional differences, genus-level relative abundance data were square-root transformed and Bray-Curtis similarity scores calculated using Primer-E (v6.1.16). Between group differences in Bray-Curtis scores were analysed using principal co-ordinate analysis (PCoA) and permutational multivariate analysis of variance (PERMANOVA) using the Primer-E PERMANOVA + add-on (v1.0.6), with 9999 permutations.

To assess taxonomic differences, genera were filtered to only those present in ≥ 10% of samples. Linear discriminant analysis (LDA) Effect Size (LEfSe) was then used to assess relative abundance differences between groups, using one-against-all multi-class analysis, and cut-offs of LDA ≥ 3 and *p* < 0.05. Wilcoxon two-sample test was used to validate LEfSe findings and compare non-parametric data between groups. Sample clustering was visualised through dendrogram of Bray-Curtis dissimilarity using the ape R package (v5.6-1). Similarity of LEfSe findings was visualised by network analysis, using Cytoscape (v3.8.1).

## Results

### Cohort characteristics

Of 170 participants, 130 (76.5%) were asthmatic. Age, gender, ethnicity, and BMI were comparable between those with and without asthma (Table 1). However, asthmatics had lower FEV_1_ (% predicted) with 70% reporting inhaled corticosteroids use in the past 12 months (Table 1).

**Table 1:** Cohort characteristics

	Asthma	No asthma
N	130	40
Female, n (%)	56 (43.1%)	20 (50.0%)
Age (years), median (IQR)	10.4 (9.23–11.8)	10.2 (8.94–11.7)
BMI (kg/m^2^), median (IQR)	18.1 (16.4–20.4)	17.1 (15.6–18.7)
Ethnicity		
European	97 (74.6%)	30 (75.0%)
Māori	23 (17.7%)	7 (17.5%)
Pacific Islander	5 (3.93%)	2 (5.0%)
Other	5 (3.93%)	2 (5.0%)
ACQ7 level, n (%)		
Well controlled	71 (54.6%)	-
Borderline	42 (32.3%)	-
Poorly controlled	17 (13.1%)	-
ICS use, past 12 months n (%)	90 (69.8%)	-
FEV_1_ (% predicted), mean (STD)	92.6 (13.6)	101.4 (11.0)
FVC (% predicted), mean (STD)	100.2 (12.1)	102.6 (9.53)
Adequate sputum quality		
Squamous cells < 30%	91 (77.8%)	26 (22.2%)
Cell viability > 50%	105 (76.1%)	33 (23.9%)
Plugs present	103 (75.7%)	33 (24.3%)
Salivary amylase < 7.5 U/mL	102 (78.5%)	28 (21.5%)

IQR: Interquartile range, STD: Standard deviation, BMI: Body mass index, ACQ7: Asthma control questionnaire, ICS: Inhaled corticosteroids, FEV_1_: Forced expiratory volume in 1 s, FVC: Forced vital capacity

### Sputum characteristics

**Fig. 1:**
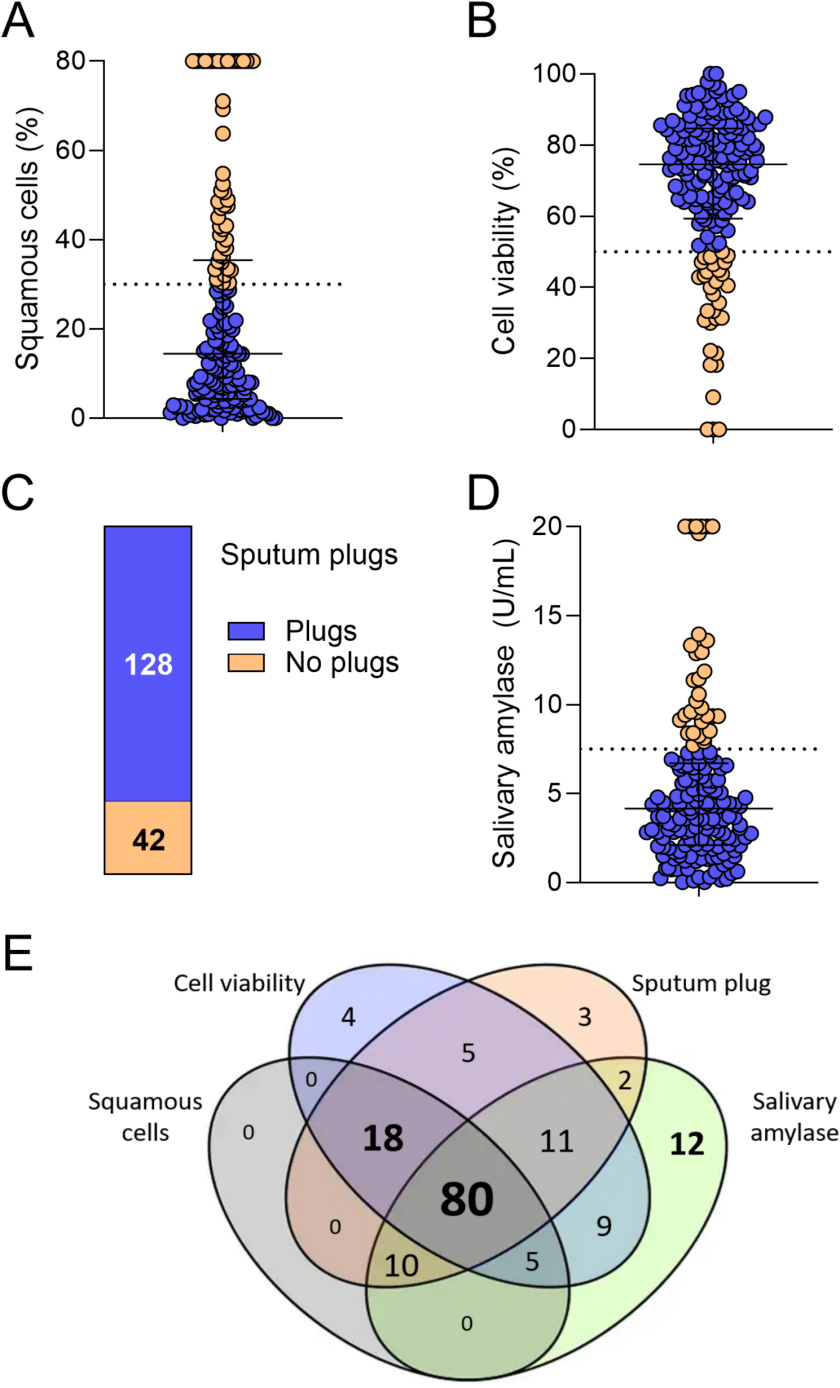
Measures of sputum acceptability. **A**) Proportion of total cell count that were squamous cells (*n* = 170), **B**) Proportion of total cell count that were viable (*n* = 170), **C**) visible sputum plugs (*n* = 170), **D**) salivary α-amylase levels (*n* = 163). **E**) Venn-diagram of number of acceptable samples overlapping across each of the four sputum quality measures (*n* = 163). Solid lines show median and interquartile range, dotted lines indicate cut-off values. Blue colour = samples that fall within the acceptable cut-off range, orange/yellow = samples that fall outside the acceptable cut-off range

Four measures of sputum quality (squamous cell%, cell viability%, sputum plugs, and salivary α-amylase), were used (Fig. 1A-D). Each of the three continuous measures (squamous cell%, salivary α-amylase and cell viability%) had a skewed distribution characterised by a long tail in the direction of poor quality/high buccal contamination. In each case, the beginning of this tail aligned well with pre-specified cut-off values (Fig. 1A, B, D). Fifty-three samples (29%) had ≥ 30% squamous cells, while 32 (19%) had < 50% cell viability, and 33 (20%) had ≥ 7.5 U/mL salivary amylase. Thirty-four samples (20%) had no visible sputum plugs. These cut-offs were subsequently utilised to define acceptable sample quality.

Alignment between the four assessed measures was high (Fig. 1E). Eighty samples (49.1%) were deemed acceptable across all 4 measures, while three (1.84%) were deemed unacceptable based on all 4 measures. Salivary α-amylase showed the poorest alignment with the three other measures, with 12 (7.3%) samples deemed acceptable based on salivary α-amylase levels, but unacceptable based on the three other measures (Fig. 1E). Similarly, 18 (11.0%) samples were not deemed acceptable by salivary α-amylase, but were acceptable by the three other measures (squamous cell%, viability, and sputum plug appearance, Fig. 1E).

The alignment of samples deemed to be of unacceptable quality (stratified by cut-off measures) with participant characteristics is presented in Supplementary Tables 1–4, with visualisation of quality markers by asthma status presented in Supplementary Fig. 1. No participant traits (either demographic or clinical) were associated with poor-quality samples.

### Poor sample quality is reflected in sputum microbiota composition

**Fig. 2:**
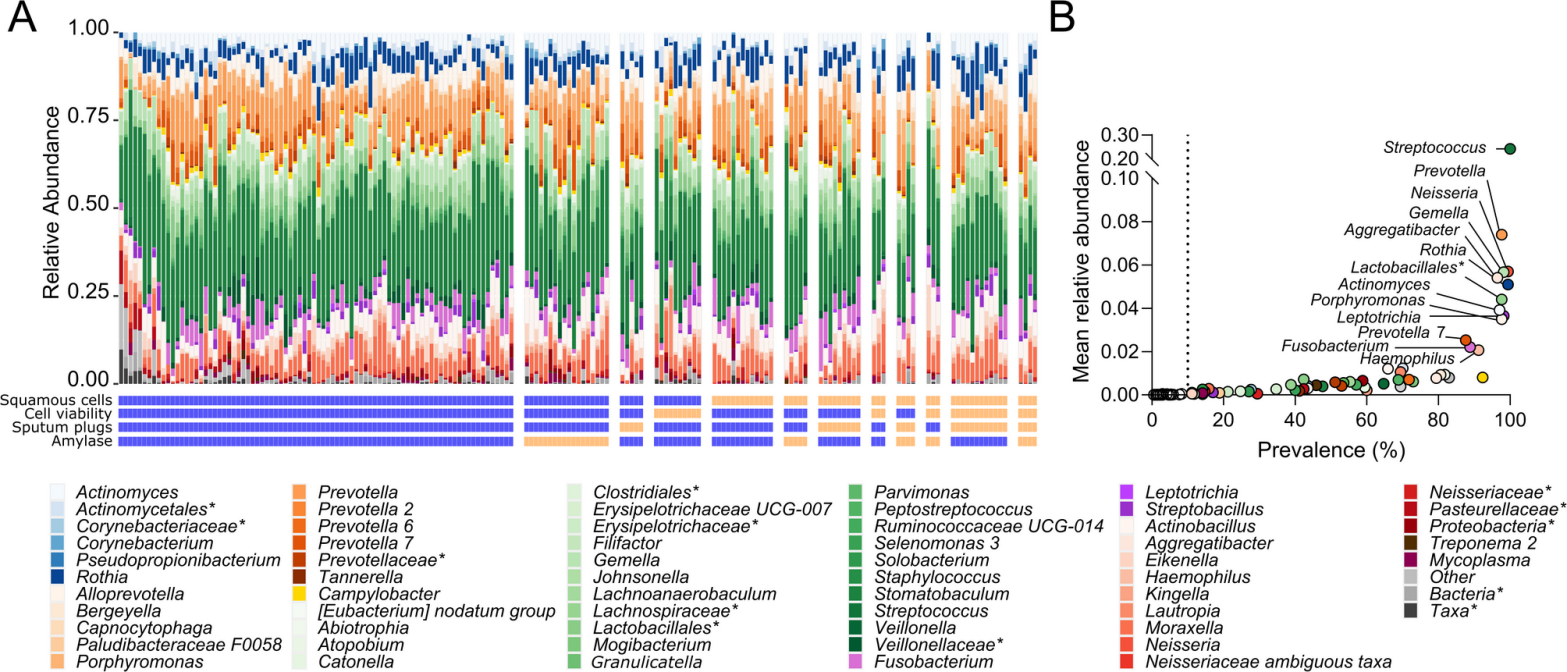
Relative abundance of genera present in ≥ 10% of samples. **A**) Taxa bar plot stratified by sputum quality where adequate quality = blue and poor quality = orange/yellow. “Other” indicates taxa present in < 10% of samples. **B**) Taxa mean relative abundance and prevalence. Genera are coloured according to phyla: Actinomycetota (formerly Actinobacteria; blue), Bacteroidota (formerly Bacteroidetes; orange), Epsilonbacteraeota (yellow), Bacillota (formerly Firmicutes; green), Fusobacteriota (formerly Fusobacteria; magenta), Pseudomonadota (formerly Proteobacteria; red), Spirochaetes (brown), Mycoplasmatota (formerly Tenericutes; crimson), Other/unclassified (grey). Asterisk (*) indicates taxa unable to be assigned to the genus level (e.g. Bacteria* indicates unassigned below the kingdom level and Taxa* indicates unassigned at below domain level)

After routine removal of reads relating to potential reagent contaminants (based on the blank extraction control), a total of 128 taxa were detected across all 170 samples. Of these, 62 were present in ≥ 10% of samples (Fig. 2A). Detected genera were primarily those common to the upper airways, including aerobes found in the oropharynx and anaerobes associated with dental plaques. *Streptococcus* had the highest mean relative abundance (24.2% ± 6.99%), followed by *Prevotella* (7.40% ± 4.09%), *Neisseria* (5.70% ± 3.00%), *Gemella* (5.67% ± 2.34%), *Aggregatibacter* (5.41% ± 2.89%), and *Rothia* (5.10% ± 3.00%, Fig. 2B). The sputum bacterial load varied, ranging from 3.2 × 10^3^ 16S copies/g to 4.1 × 10^7^16S copies/g (median = 2.8 × 10^6^ (interquartile range: 1.3 × 10^6^ to 6.1 × 10^6^) 16S copies/g.

**Fig. 3:**
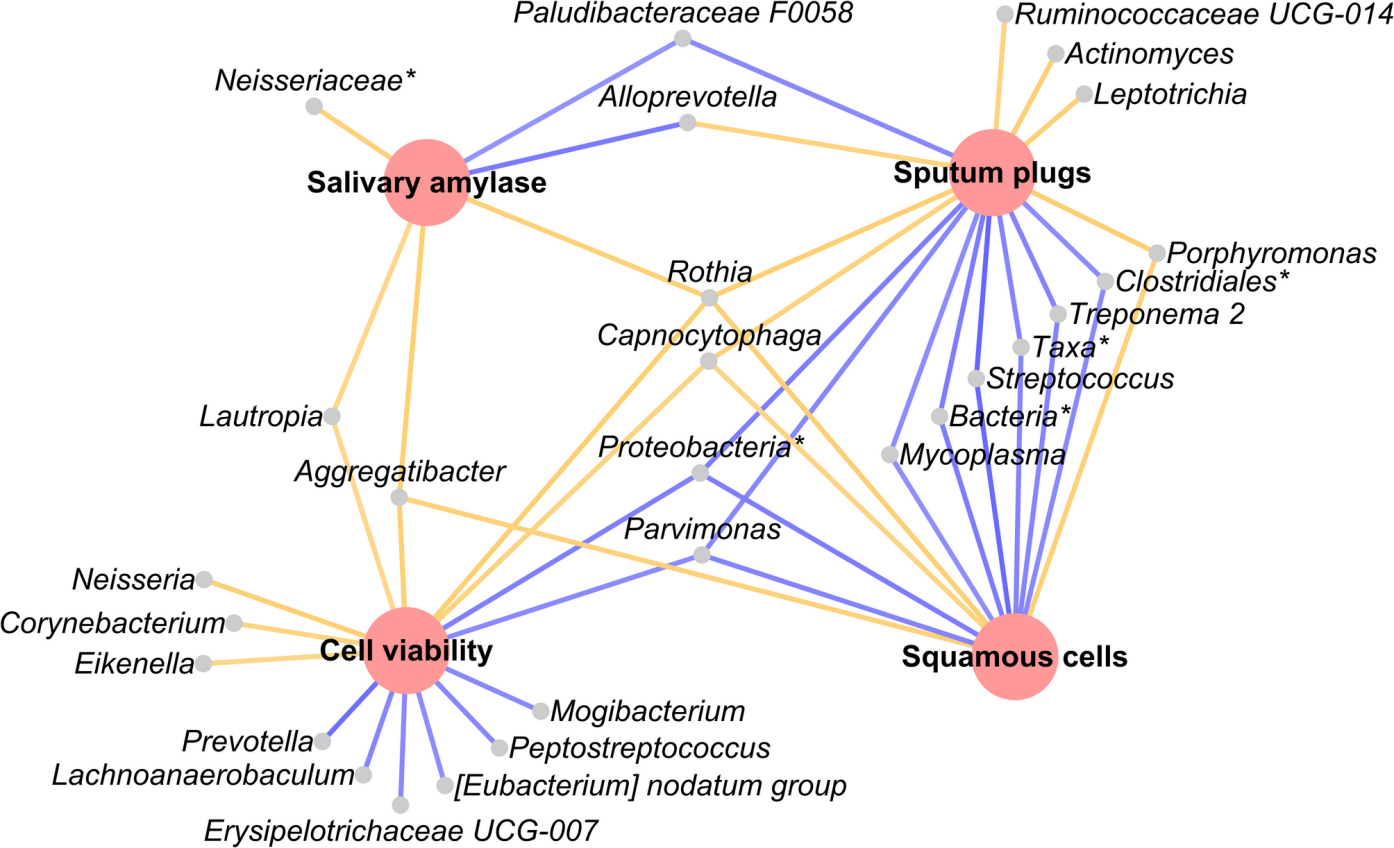
Taxa that differed significantly based on sputum acceptability criteria. Large red nodes = sputum acceptability criteria: salivary amylase (cut-off of 7.5 U/mL), sputum plugs (visible or not visible), cell viability (cut-off 50% viable), and squamous cells (cut-off of 30%). Small grey nodes = taxa. Orange/yellow line = taxa is higher in samples with poor acceptability. Blue line = taxa is higher in samples with adequate acceptability. Asterisk (*) indicates taxa unable to be assigned to the genus level (e.g. Bacteria* indicates unassigned below the kingdom level and Taxa* indicates unassigned at below domain level). Statistics: Linear discriminant analysis (LDA) Effect Size (LEfSe)

Stratification by quality based on any of the four quality criteria identified significant divergence of sputum microbiota between samples deemed acceptable and unacceptable (*p* ≤ 0.05, Supplementary Fig. 2). Divergence between these two groups was greatest when samples were stratified according to presence or absence of visible sputum plugs (pseudo-F = 3.22), followed by squamous cell% (pseudo-F = 3.13), cell viability% (pseudo-F = 2.52), and salivary amylase (pseudo-F = 1.75) (Supplementary Table 5). Bacterial taxa that differed between groups for each sputum quality measure are shown in Fig. 3 and Supplementary Fig. 3. Notably, *Rothia* was more abundant in poor quality samples, regardless of the measure used, while stratification by squamous cell% or sputum plugs identified higher levels of *Streptococcus*,* Treponema* 2, and sequences unassigned to any taxa in adequate quality samples.

Total bacterial load was also significantly associated with sputum quality. Specifically, samples with acceptable squamous cell% had a significantly lower bacterial load compared to unacceptable samples (*p* = 0.0057, Supplementary Fig. 4). However, bacterial load was not significantly different when stratified according to cell viability%, sputum plugs, or salivary α-amylase level (*p* > 0.05, Supplementary Fig. 4).

### Sputum microbiota is associated with asthma following removal of low-quality samples

**Fig. 4:**
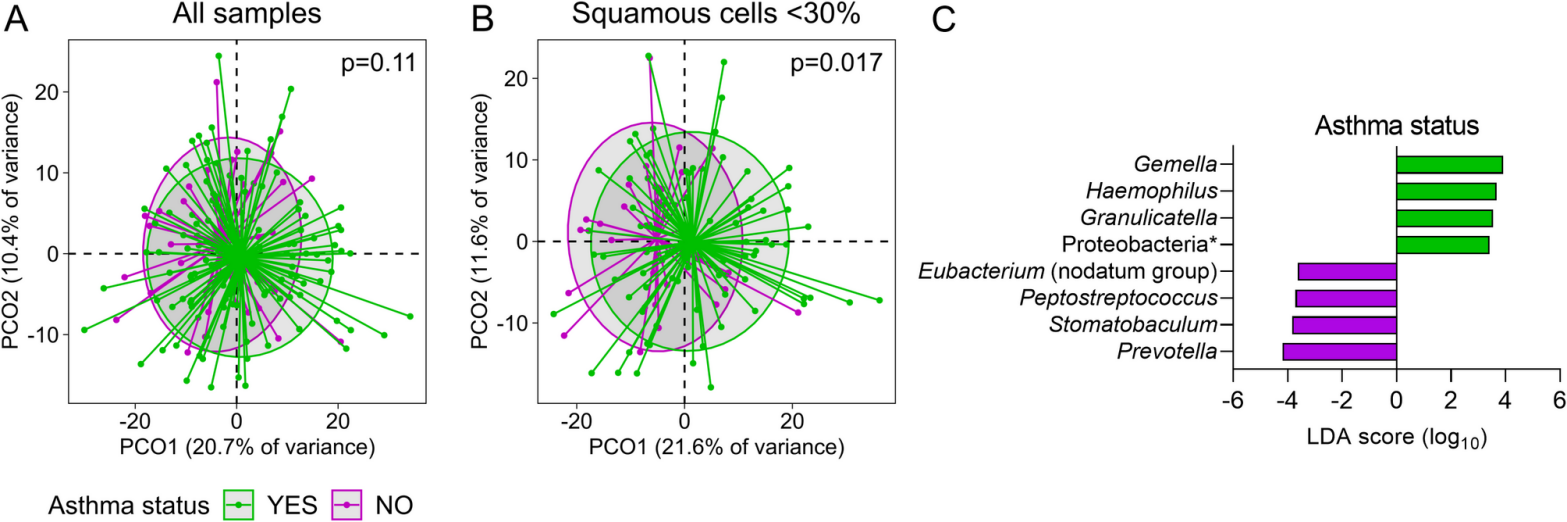
Sputum microbiota differs between asthma (green) and no asthma (purple) following exclusion of poor-quality samples. **A**) Principal coordinate analysis (PCoA) of all 170 samples. **B**) PCoA of the 117 samples with < 30% squamous cells. **C**) Taxa that differed significantly based on asthma status in samples with < 30% squamous cells. Statistics: **A** and **B**) permutational multivariate analysis of variance (PERMANOVA) and **C**) Linear discriminant analysis (LDA) Effect Size (LEfSe)

When samples from all 170 participants were included in the analysis, no significant differences in sputum microbiota characteristics could be identified between individuals with and without asthma (pseudo-F = 1.46, *p* = 0.11, Fig. 4A, Supplementary Table 6). In contrast, the exclusion of 53 samples based on a 30% squamous cell cut-off resulted in a significant separation of microbiota composition between those with and without asthma (pseudo-F = 2.07, *p* = 0.017, Fig. 4B, Supplementary Table 6). Exploration of taxa that explained this difference revealed the opportunistic pathobiont genus *Haemophilus*, along with *Gemella*, and *Granulicatella* as significantly higher in asthma (Fig. 4C). In contrast, when poor quality samples are included, fewer taxa overall differed between those with and without asthma (Supplementary Fig. 5), with *Haemophilus* levels being comparable (*p* = 0.26).

Exclusion of poor quality samples using any of the other three measures revealed no separation of the microbiota between those with and without asthma (Supplementary Table 6). However, a significant divergence in microbiota between asthma and no asthma remained when combinations of cut-offs were utilised that included squamous cell%, (Supplementary Table 6), even in sensitivity analyses where lower squamous cell% cut-offs (20 and 25%) were used (Supplementary Table 6).

## Discussion

The microbial content of sputum samples is generally considered to be representative of the lower and central airways [29]. However, transit of sputum through the upper respiratory tract and mouth results in contamination by upper airways and buccal cavity microbes [7, 10, 30, 31]. Other sampling methods, such as bronchoalveolar lavage and bronchial brushings can better avoid buccal contamination, but are more invasive. Our goal was to determine whether removing sputum samples identified empirically as low quality increased our ability to identify differences in sputum microbiota characteristics between children with and without asthma.

Our analysis involved the application of three commonly measured sputum quality variables that require application to fresh samples (squamous cell%, cell viability%, presence of visible sputum plugs) and one that has the potential to be applied to stored material (salivary α-amylase). The four assessed quality variables showed broad alignment, with salivary α-amylase showing the lowest congruence. Stratification of sputum microbiota on the basis of these sputum quality variables identified significant microbiota divergence between samples deemed to be of acceptable and unacceptable quality. Poor sample quality was characterised by higher bacterial load and relative abundance of oropharyngeal taxa. The higher bacterial load in poor quality samples likely reflects increased contamination by buccal-derived bacteria, which has a higher bacterial load compared to the lower airways [7]. Further, removal of poor quality samples based on a squamous cell percentage cut-off revealed significant differences in sputum microbiota composition between asthmatic and non-asthmatic children that were otherwise not detectable.

The proportion of squamous and non-squamous human cells in sputum samples (representing the upper and lower airways respectively) is a well-established basis for determining sample origin [1], while cell viability is used primarily to determine whether samples are of sufficient quality for downstream analysis [1]. These measures are commonly standardised [1] and show high reproducibility between centres [32]. In addition, visual assessment of sputum provides a semi-quantifiable measure of quality. Clearly visible sputum plugs in a selected sample are indicative of material originating in the lower airways, while large amounts of saliva, particularly in the absence of plugs, suggests substantial contribution of material from the upper airways/buccal cavity. Finally, while not assessed routinely, salivary α-amylase measurement represents a novel approach to quantifying the contribution of oral secretions to sputum samples, and unlike other measures, may be applicable to both fresh and frozen samples. However, while there was some similarity between salivary α-amylase levels and the other three measures assessed, overall, salivary α-amylase was a relatively poor marker of sputum quality. This is likely to reflect the high variability in salivary α-amylase levels, both temporally [33], and between individuals [34].

We found that the exclusion of samples with ≥ 30% squamous cells resulted in a significant divergence in airway microbiota composition between children with and without asthma. This finding remained true when modifying the squamous cell % cut-off to 20% and 25%, as well as including single combinations of the other quality markers (cell viability, plugs and salivary amylase), indicating squamous cell % as the primary discriminant quality marker in this study. Importantly, exclusion of poor quality samples was characterised by higher levels of *Haemophilus* in asthmatic children. *Haemophilus* (in particular *H. influenzae*) appears to be a central taxon associated with both asthma development and asthma severity [14, 21, 35].

While the utility and potential benefit of targeting airway microbiology to improve clinical outcomes in asthma remains unclear, a *post hoc* analysis of a randomised controlled trial of adults with difficult-to-treat asthma identified *H. influenzae* abundance in the airways as a predictor of response to long-term, low-dose azithromycin therapy [36]. The extent to which *Haemophilus* is associated with treatment outcomes in children is not yet known, but it is possible that microbiota assessment in childhood asthma may inform and guide treatment in difficult-to-treat disease.

We chose to exclude samples that, based on the quality criteria selected, poorly represented the lower airways. Such an approach, while reducing the effect of upper airway contamination on analyses, also results in a reduced study population, with implications for statistical power. Further, exclusion of lower quality samples may result in selection for more productive individuals, or for those with more mucopurulent or inflammatory disease. While we did not identify differences in cohort characteristics following the application of sputum quality thresholds, such an effect cannot be ruled out. An alternative strategy, involving the in silico post-sequencing removal of reads likely to have originated from upper airway microbes [37], has been proposed, potentially allowing greater sample retention. However, this approach was developed in the context of adult cystic fibrosis, where lower airway bacterial biomass is commonly high, reducing the impact of salivary contamination. Such an approach also requires the parallel analysis of paired saliva samples and risks the removal of reads from bacteria that are found in the upper airways, but which are also capable of colonising the lungs.

This study has several limitations. Firstly, upper airway contamination was characterised using commonly applied markers of sputum quality. To assess comprehensively whether sputum quality markers accurately reflect the upper versus lower airways, alignment with paired samples from the upper and lower airways would be required (for example, mouthwash samples and bronchial brushings or bronchoalveolar lavage). These were not available. Secondly, a single induced sputum sample was utilised per participant for this analysis. Serial sampling of sputum has been shown to provide a more complete microbiota profile [38] as well as inflammatory phenotype stability [39]. Thirdly, our study focused on induced sputum from children with and without asthma. The wider applicability of the measures and thresholds employed in the context of other diseases and sample types (e.g. spontaneous sputum) is not known. Finally, we tested sputum quality cut-offs that were determined a priori, guided by the literature. We did not seek to identify the most appropriate cut-off value. However, for sensitivity testing, we explored two other common squamous cell cut-off percentages (≥ 20% and ≥ 25%) which also showed a significant divergence in sputum microbiota between asthma and controls, despite resulting in the exclusion of a greater number of samples (*n* = 8 and 16 samples respectively).

In summary, we report an association between childhood asthma and sputum microbiota characteristics that is apparent only following the objective removal of poor-quality samples based on a ≥ 30% squamous cell cut-off. Adaptation of this approach to other respiratory contexts has the potential to provide considerable benefit, including in identifying subtle treatment effects and discriminating between closely related disease phenotypes.

## Electronic supplementary material

Below is the link to the electronic supplementary material.

Supplementary Material 1

Supplementary Material 2

## Data Availability

Sequencing data have been deposited in the European Bioinformatics Institute European Nucleotide Archive (PRJEB57744). The STORMS Microbiome Reporting Checklist [40] is available in Supplementary Table 7.
