# Supplementary material for: Sputum quality affects assessment of airway microbiology in childhood asthma
Source: Respir Res. 2025 Jun 4;26:209. doi: 10.1186/s12931-025-03266-x (PMC12139374; doi:10.1186/s12931-025-03266-x)
Supplement: Supplementary file 2 — Supplementary Material 2 [file 12931_2025_3266_MOESM2_ESM.docx]

**SUPPLEMENTARY METHODS**

***Recruitment and clinical assessments***

All participants completed a respiratory questionnaire based on the International Study of Asthma and Allergies in Childhood (ISAAC) Phase II survey (1). Asthma control was assessed using the Asthma Control Questionnaire (ACQ7) (2). Participants with a respiratory infection or asthma exacerbation within 1 month of assessment returned when symptom-free and those with FEV_1_% predicted <75% were excluded. Prior to testing, asthma medication and antihistamines were withheld for ≥12 and ≥24 hours, respectively.

***Combined hypertonic saline challenge and sputum induction***

Aerosolised hypertonic saline (4.5% w/v) was produced using an ultrasonic nebuliser (DeVilbiss Ultraneb 2000, Langen, Germany) and administered orally through a mouthpiece (Hans-Rudolph Inc, Kansas City, USA) for increasing intervals from 0.5-4 minutes, to a total of 16 minutes. Spirometry was conducted between intervals, and salbutamol was administered if FEV_1_ dropped to ≤75%-predicted. Participants were subsequently encouraged to produce sputum in a sterile plastic container.

***Sputum DNA extraction***

Sputum processing was performed at the South Australian Health and Medical Research Institute. Following removal of supernatant, sputum pellets had 300 μL of Tris-EDTA solution (10 mM Tris-HCl, 1 mM EDTA; pH 8.0; Ambion, ThermoFisher Scientific, Victoria, Australia), 200 mg of silica: zirconium beads (1:1 of 0.1 μm and 1.0 μm; Biospec Products, Inc., OK, USA), and a single chrome bead (3.2 mm, Biospec Products, Inc., OK, USA) added. Samples underwent bead-beating at 6.5 m/s for 60 sec in a FastPrep®-24 Instrument (MP Biomedicals, CA, USA). Homogenised samples were heated to 95 °C for 1 min, before being cooled on ice for 1 min. Lysozyme (ROCHE, ThermoFisher Scientific, Victoria, Australia) and lysostaphin (Sigma-Aldrich, MO, USA) were then added to a final concentration of 2 mg/mL and 0.1 mg/mL, respectively, and samples incubated at 37 °C for 1 hr. Proteinase K (Fermentas, ThermoFisher Scientific, Victoria, Australia) and sodium dodecyl sulphate (Sigma-Aldrich, MO, USA) were then added to a final concentration of 1.2 mg/mL and 1.5 %, w/v, respectively. Following incubation at 30 min at 56 °C, 40 μL of 5 M sodium chloride and 450 μL of phenol:chloroform:isoamyl alcohol (25:24:1; saline buffered at pH8.0; Sigma-Aldrich, MO, USA) were added and samples vortexed for 30 sec. The aqueous-organic layers were separated by centrifugation at 13,000 × g for 10 min at 4 °C and 400 μL of the aqueous layer was transferred to a new microfuge tube. DNA was recovered using an EZ-10 Spin column in accordance with manufacturer’s instructions (Bio Basic, Inc., Ontario, Canada), following precipitation by the addition of 10 M ammonium acetate and 99% ethanol (Sigma Aldrich, MO, USA) in a 1:10 and 1:1 ratio with sample volume, respectively. DNA was eluted in 100 μL UltraPure DNase/RNase-free distilled water (Gibco, ThermoFisher Scientific, Victoria, Australia) and stored at -80 °C prior to analysis.

***Total bacterial load qPCR***

Total bacterial load was approximated by measuring the 16S rRNA gene copy number by quantitative PCR (qPCR). The qPCR was performed as previously described (3-5), with the universal 16S rRNA primers: B331F (5'-TCCTACGGGAGGCAGCAGT-3') and B797R (5'-GGACTACCAGGGTATCTAATCCTGTT-3'), on the QuantStudio 6 Flex System (Thermo Fisher Scientific, Vic, Australia). Reactions were performed in triplicate and averages taken. The 16S rRNA copy number was calculated against a standard curve of a known bacterial concentration and normalised per g of sputum.

***16S rRNA gene amplicon sequencing***

The V1-3 hypervariable region of the bacterial 16S rRNA gene was amplified from sputum DNA using modified primers 27F (5'-TCGTCGGCAGCGTCAGATGTGTATAAGAGACAGAGRGTTTGATCMTGGCTCAG-3') and 519R (5'-GTCTCGTGGGCTCGGAGATGTGTATAAGAGACAGGTNTTACNGCGGCKGCTG-3'), with Illumina adapter overhang sequences as indicated by underline. Amplicons were generated, cleaned, indexed and sequenced according to the Illumina MiSeq 16S Metagenomic Sequencing Library Preparation protocol with certain modifications. Briefly, an initial PCR reaction contained at least 12.5 ng of DNA, 5 μL of forward primer (1 μM), 5 μL of reverse primer (1 μM) and 12.5 μL of 2 × KAPA HiFi Hotstart ReadyMix (KAPA Biosystems, Wilmington, MA, USA) in a total volume of 25 μL. The PCR reaction was performed on a Veriti 96-well Thermal Cycler (Life Technologies) using the following program: 95 °C for 3 min, followed by 25 cycles of 95 °C for 30 sec, 55 °C for 30 sec and 72 °C for 30 sec and a final extension step at 72 °C for 5 min. Samples were multiplexed using a dual-index approach with the Nextera XT Index kit (Illumina Inc., San Diego, CA, USA) according to the manufacturer’s instructions. The final library was paired-end sequenced at 2 × 300 bp using a MiSeq Reagent Kit v3 on the Illumina MiSeq platform. Sequencing was performed at the David R Gunn Genomics Facility, South Australian Health and Medical Research Institute. Reads have been deposited in the European Bioinformatics Institute European Nucleotide Archive (PRJEB57744).

***Bioinformatic processing***

Sequence output was demultiplexed using QIIME2 (6)(release 2019.4). The DADA2 plugin was used to trim, de-replicate, merge, and remove chimeric sequences, as well as identify and correct sequencing errors (7). Representative sequences were aligned to the SILVA database (v132) at 80% using vsearch and unassigned sequences were filtered out. Remaining unique amplicon sequence variants (ASVs) were classified using the QIIME2 sklearn algorithm to the SILVA database at 99% sequence similarity. ASVs that were amplified in the blank extraction control were examined. Contaminant or spurious taxa, identified in the blank and not associated with the human microbiota were filtered out. This represented an average of 2.23% of reads ±1.86% belonging primarily to the Family Comamonadaceae, including *Rhizobium*, *Sphingomonas, Variovorax*, *Acidovorax*, and *Comamonas.*

**Supplementary Table 1**: Participant demographics stratified by sputum squamous cells %

|  | Squamous <30% | Squamous ≥30% |
| --- | --- | --- |
| N | 117 | 53 |
| Female, n (%) | 48 (41.0%) | 28 (52.8%) |
| Age (years), median (IQR) | 10.3 (9.06-11.7) | 10.6 (9.19-11.9) |
| BMI, median (IQR) | 17.9 (16.4-20.2) | 18.0 (16.0-20.3) |
| Ethnicity |  |  |
| European | 88 (75.2%) | 39 (73.6%) |
| Māori | 19 (16.2%) | 11 (20.8%) |
| Pacific Islander | 5 (4.3%) | 1 (1.9%) |
| Other | 5 (4.3%) | 2 (3.8%) |
| Asthma, n (%) | 91 (77.8%) | 39 (73.6%) |
| ACQ level, n (%) |  |  |
| Well controlled | 49 (53.8%) | 22 (56.4%) |
| Borderline | 31 (34.1%) | 11 (28.2%) |
| Poorly controlled | 11 (12.1%) | 6 (15.4%) |
| ICS use, n (%) | 60 (65.6%) | 30 (76.9%) |
| FEV_1_ %, mean (STD) | 95.1 (13.2) | 93.7 (14.3) |
| FVC %, mean (STD) | 101.2 (10.9) | 99.7 (12.8) |

IQR: Interquartile range, STD: Standard deviation, BMI: Body mass index, ACQ7: Asthma control questionnaire, ICS: Inhaled corticosteroids, FEV_1_: Forced expiratory volume in 1 second, FVC: Forced vital capacity

**Supplementary Table 2**: Participant demographics stratified by sputum cell viability

|  | Cell viability ≥50% | Cell viability <50% |
| --- | --- | --- |
| N | 138 | 32 |
| Female, n (%) | 60 (43.5%) | 16 (50.0%) |
| Age (years), median (IQR) | 10.3 (8.94-11.5) | 11.0 (9.51-12.0) |
| BMI, median (IQR) | 17.8 (16.2-20.1) | 18.2 (16.1-20.5) |
| Ethnicity |  |  |
| European | 103 (74.6%) | 24 (75.0%) |
| Māori | 25 (18.1%) | 5 (15.6%) |
| Pacific Islander | 5 (3.6%) | 1 (3.1%) |
| Other | 5 (3.6%) | 2 (6.3%) |
| Asthma, n (%) | 105 (76.1%) | 25 (78.1%) |
| ACQ level, n (%) |  |  |
| Well controlled | 58 (54.7%) | 14 (56.0%) |
| Borderline | 35 (33.0%) | 7 (28.0%) |
| Poorly controlled | 13 (12.3%) | 4 (16.0%) |
| ICS use, n (%) | 71 (68.3%) | 19 (73.1%) |
| FEV_1_ %, mean (STD) | 95.3 (13.2) | 91.8 (14.5) |
| FVC %, mean (STD) | 101.1 (11.6) | 99.1 (11.5) |

IQR: Interquartile range, STD: Standard deviation, ACQ7: Asthma control questionnaire, ICS: Inhaled corticosteroids, FEV_1_: Forced expiratory volume in 1 second, FVC: Forced vital capacity

**Supplementary Table 3**: Participant demographics stratified by visible sputum plugs

|  | Sputum plugs | No sputum plugs |
| --- | --- | --- |
| N | 136 | 34 |
| Female, n (%) | 56 (41.2%) | 20 (58.8%) |
| Age (years), median (IQR) | 10.3 (9.13-11.6) | 11.2 (9.11-12.0) |
| BMI, median (IQR) | 17.9 (16.2-20.3) | 17.8 (16.0-20.3) |
| Ethnicity |  |  |
| European | 101 (74.3%) | 26 (76.5%) |
| Māori | 24 (17.7%) | 6 (17.7%) |
| Pacific Islander | 5 (3.7%) | 1 (2.9%) |
| Other | 6 (4.4%) | 1 (2.9%) |
| Asthma, n (%) | 103 (75.7%) | 27 (79.4%) |
| ACQ level, n (%) |  |  |
| Well controlled | 58 (55.8%) | 14 (51.9%) |
| Borderline | 33 (31.7%) | 9 (33.3%) |
| Poorly controlled | 13 (12.5%) | 4 (14.8%) |
| ICS use, n (%) | 71 (68.9%) | 19 (70.4%) |
| FEV_1_ %, mean (STD) | 95.6 (13.6) | 90.7 (12.6) |
| FVC %, mean (STD) | 101.4 (11.3) | 98.3 (12.4) |

IQR: Interquartile range, STD: Standard deviation, ACQ7: Asthma control questionnaire, ICS: Inhaled corticosteroids, FEV_1_: Forced expiratory volume in 1 second, FVC: Forced vital capacity

**Supplementary Table 4**: Participant demographics stratified by salivary amylase level

|  | Amylase <7.5 U/mL | Amylase ≥7.5 U/mL |
| --- | --- | --- |
| N^*^ | 130 | 33 |
| Female, n (%) | 55 (42.3%) | 18 (54.6%) |
| Age (years), median (IQR) | 10.4 (8.91-11.7) | 10.8 (9.53-11.6) |
| BMI, median (IQR) | 18.0 (16.0-20.3) | 17.6 (16.8-19.3) |
| Ethnicity |  |  |
| European | 100 (76.9%) | 22 (66.7%) |
| Māori | 23 (17.7%) | 6 (18.2%) |
| Pacific Islander | 2 (1.5%) | 3 (9.1%) |
| Other | 5 (3.9%) | 2 (6.1%) |
| Asthma, n (%) | 102 (78.5%) | 23 (69.7%) |
| ACQ level, n (%) |  |  |
| Well controlled | 58 (56.3%) | 10 (43.5%) |
| Borderline | 33 (32.0%) | 9 (39.1%) |
| Poorly controlled | 12 (11.7%) | 4 (17.4%) |
| ICS use, n (%) | 68 (66.7%) | 18 (78.3%) |
| FEV_1_ %, mean (STD) | 94.8 (13.3) | 94.2 (14.2) |
| FVC %, mean (STD) | 101.1 (11.5) | 99.9 (12.3) |

IQR: Interquartile range, STD: Standard deviation, ACQ7: Asthma control questionnaire, ICS: Inhaled corticosteroids, FEV_1_: Forced expiratory volume in 1 second, FVC: Forced vital capacity. ^*^7 samples were unavailable for salivary amylase analysis

**Supplementary Table 5:** Difference in microbiota beta-diversity using different sputum quality criteria. Performed using permutational multivariate analysis of variance (PERMANOVA).

|  | N | Pseudo-F | R^2^ (%) | P(perm) |
| --- | --- | --- | --- | --- |
| Squamous cell (30% cut-off) | 170 | 3.13 | 1.83 | **0.0012** |
| Cell viability (50% cut-off) | 170 | 2.52 | 1.48 | **0.0046** |
| Sputum plugs visible | 170 | 3.22 | 1.88 | **0.0007** |
| Salivary amylase (7.5 U/mL cut-off) | 163 | 1.75 | 1.07 | **0.049** |

**Supplementary Table 6:** Microbiota beta-diversity difference by asthma status including only samples of acceptable quality based on quality measures. Performed using permutational multivariate analysis of variance (PERMANOVA).

|  | **N** | **Pseudo-F** | **R^2^ (%)** | **P(perm)** |
| --- | --- | --- | --- | --- |
| All samples | 170 | 1.46 | 0.86 | 0.11 |
| **Individual sputum quality assessments** | | | | |
| Squamous cell <30% | 117 | 2.07 | 1.77 | **0.017** |
| Cell viability ≥50% | 138 | 1.53 | 1.11 | 0.097 |
| Sputum plugs visible | 136 | 1.37 | 1.01 | 0.15 |
| Salivary amylase <7.5 U/mL | 130 | 1.23 | 0.95 | 0.23 |
| **Sputum quality combinations** | | | | |
| Squamous cell <30% + Cell viability ≥50% | 107 | 2.33 | 2.17 | **0.011** |
| Squamous cell <30% + visible plugs | 112 | 1.76 | 1.57 | **0.040** |
| Squamous cell <30% + salivary amylase <7.5 U/mL | 95 | 1.88 | 1.99 | **0.033** |
| Cell viability ≥50% + visible plugs | 120 | 1.50 | 1.25 | 0.10 |
| Cell viability ≥50% + salivary amylase <7.5 U/mL | 105 | 1.47 | 1.40 | 0.11 |
| Visible plugs + Salivary amylase <7.5 U/mL | 104 | 1.20 | 1.17 | 0.24 |
| Squamous cell <30% + visible plugs + cell viability ≥50% | 102 | 2.03 | 1.99 | **0.022** |
| **Sensitivity analysis of different squamous cell % cut-off values** | | | | |
| Squamous cell <25% | 109 | 2.35 | 2.15 | **0.0092** |
| Squamous cell <20% | 101 | 2.11 | 2.09 | **0.018** |

**Supplementary Figure 1:** Sputum acceptability cut-offs between individuals with and without asthma. A) Proportion of total cell count that were squamous cells (n=170), B) Proportion of total cell count that were viable (n=170), C) visible sputum plugs (n=170), D) salivary α-amylase levels (n=163). Blue colour = samples that fall within the acceptable cut-off range, orange/yellow = samples that fall outside the acceptable cut-off range.

**
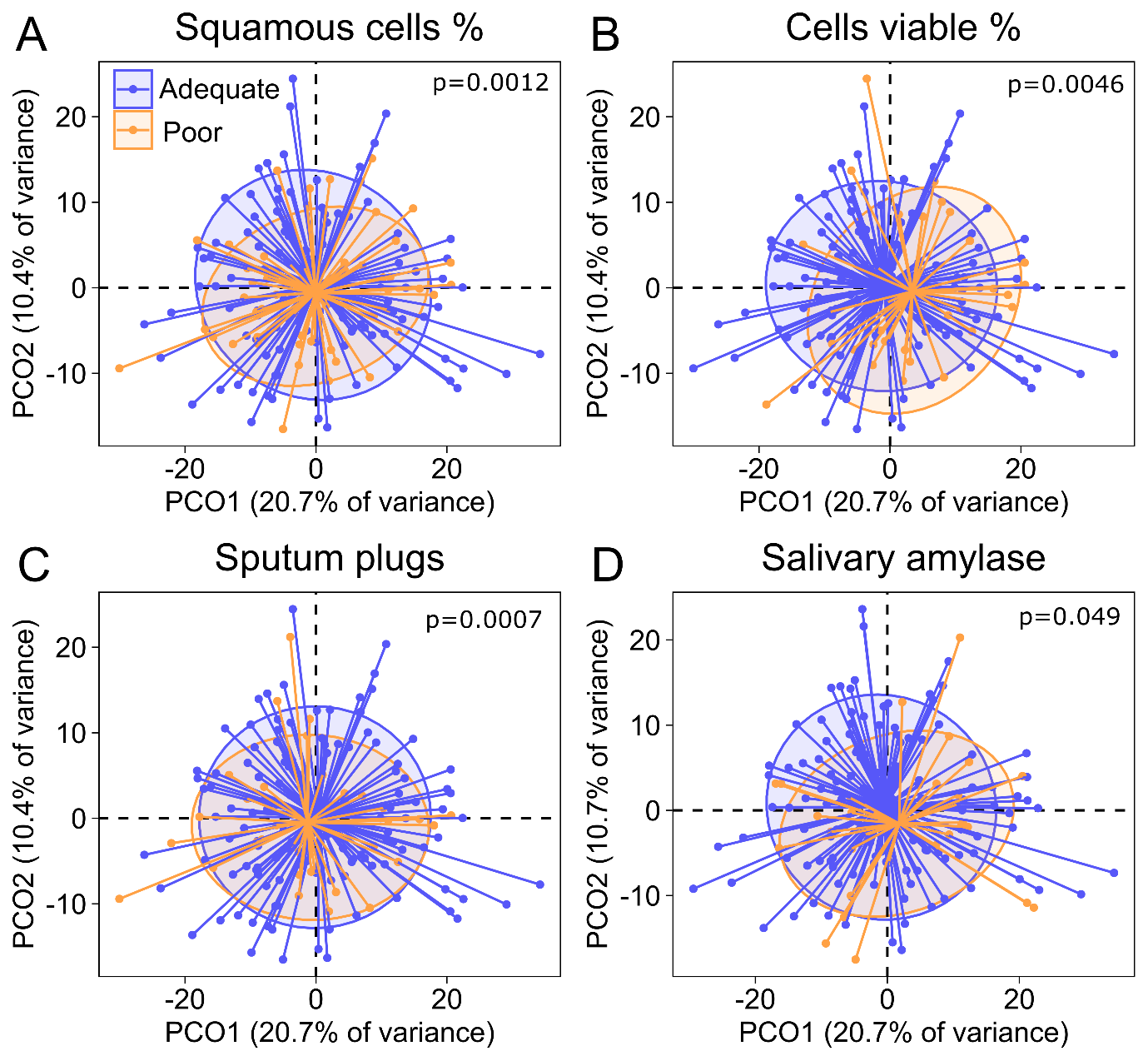
 Supplementary Figure 2:** Sputum microbiota differs by sample acceptability cut-offs. Principle coordinate analysis (PCoA) of sputum microbiota coloured by acceptability criteria. A) squamous cell % (cut-off of 30%), B) cell viability % (cut-off 50% viable), C) sputum plugs (visible or not visible), and D) salivary α-amylase activity (cut-off of 7.5 U/mL). Statistics: permutational multivariate analysis of variance.

**Supplementary Figure 3**: Taxa that differed significantly based on sputum acceptability criteria. A) squamous cell % (cut-off 30%), B) cell viability % (cut-off 50%), C) sputum plugs (visible/not visible), and D) salivary α-amylase activity (cut-off 7.5 U/mL). Yellow = taxa is higher in samples with poor acceptability. Blue = taxa is higher in samples with adequate acceptability. Asterisk (*) indicates taxa unable to be assigned to the genus level (e.g. Bacteria* indicates unassigned below the kingdom level and Taxa* indicates unassigned at below domain level). Statistics: Linear discriminant analysis (LDA) Effect Size (LEfSe).

**Supplementary Figure 4:** Total bacterial load stratified by sample quality criteria. A) Squamous cell % cut-off = 30%; B) cell viability % cut-off = 50%; C) sputum plug cut-off = adequate (visible); D) salivary α-amylase cut-off = 7.5 U/mL. Statistics: Mann-Whitney U test.

**Supplementary Figure 5:** Analysis of taxa by asthma when all samples included. A) Taxa that differed significantly between asthma (green) and no asthma (purple). B) *Haemophilus* relative abundance. Asterisk (*) indicates taxa unable to be assigned to the genus level. Statistics: A) Linear discriminant analysis (LDA) Effect Size (LEfSe); B) Mann-Whitney U test.

**References**

1. Weiland SK, Bjorksten B, Brunekreef B, Cookson WO, von Mutius E, Strachan DP, et al. Phase II of the International Study of Asthma and Allergies in Childhood (ISAAC II): rationale and methods. The European respiratory journal. 2004;24(3):406-12.

2. Juniper EF, O'Byrne PM, Guyatt GH, Ferrie PJ, King DR. Development and validation of a questionnaire to measure asthma control. The European respiratory journal. 1999;14(4):902-7.

3. Taylor SL, Leong LEX, Choo JM, Wesselingh S, Yang IA, Upham JW, et al. Inflammatory phenotypes in patients with severe asthma are associated with distinct airway microbiology. The Journal of allergy and clinical immunology. 2018;141(1):94-103 e15.

4. Taylor SL, Leong LEX, Ivey KL, Wesselingh S, Grimwood K, Wainwright CE, et al. Total bacterial load, inflammation, and structural lung disease in paediatric cystic fibrosis. Journal of cystic fibrosis : official journal of the European Cystic Fibrosis Society. 2020;19(6):923-30.

5. Nadkarni MA, Martin FE, Jacques NA, Hunter N. Determination of bacterial load by real-time PCR using a broad-range (universal) probe and primers set. Microbiology. 2002;148(1):257-66.

6. Bolyen E, Rideout JR, Dillon MR, Bokulich NA, Abnet CC, Al-Ghalith GA, et al. Reproducible, interactive, scalable and extensible microbiome data science using QIIME 2. Nat Biotechnol. 2019;37(8):852-7.

7. Callahan BJ, McMurdie PJ, Rosen MJ, Han AW, Johnson AJ, Holmes SP. DADA2: High-resolution sample inference from Illumina amplicon data. Nature methods. 2016;13(7):581-3.
